# Surface Deformation of Biocompatible Materials: Recent Advances in Biological Applications

**DOI:** 10.3390/biomimetics9070395

**Published:** 2024-06-28

**Authors:** Sunhee Yoon, Ahmed Fuwad, Seorin Jeong, Hyeran Cho, Tae-Joon Jeon, Sun Min Kim

**Affiliations:** 1 Department of Biological Sciences and Bioengineering, Inha University, 100, Inha-ro, Michuhol-gu, Incheon 22212, Republic of Korea; yoonsh0912@inha.ac.kr (S.Y.); hrcho314@gmail.com (H.C.); 2 Industry-Academia Interactive R&E Center for Bioprocess Innovation (BK21), Inha University, 100, Inha-ro, Michuhol-gu, Incheon 22212, Republic of Korea; 3 Department of Mechanical Engineering, Inha University, 100, Inha-ro, Michuhol-gu, Incheon 22212, Republic of Korea; ahmed@inha.ac.kr (A.F.); tjfls0923@gmail.com (S.J.); 4 Biohybrid Systems Research Center, Inha University, 100, Inha-ro, Michuhol-gu, Incheon 22212, Republic of Korea

**Keywords:** surface deformation, surface topography, substrates, cell chip, biosensors

## Abstract

The surface topography of substrates is a crucial factor that determines the interaction with biological materials in bioengineering research. Therefore, it is important to appropriately modify the surface topography according to the research purpose. Surface topography can be fabricated in various forms, such as wrinkles, creases, and ridges using surface deformation techniques, which can contribute to the performance enhancement of cell chips, organ chips, and biosensors. This review provides a comprehensive overview of the characteristics of soft, hard, and hybrid substrates used in the bioengineering field and the surface deformation techniques applied to the substrates. Furthermore, this review summarizes the cases of cell-based research and other applications, such as biosensor research, that utilize surface deformation techniques. In cell-based research, various studies have reported optimized cell behavior and differentiation through surface deformation, while, in the biosensor and biofilm fields, performance improvement cases due to surface deformation have been reported. Through these studies, we confirm the contribution of surface deformation techniques to the advancement of the bioengineering field. In the future, it is expected that the application of surface deformation techniques to the real-time interaction analysis between biological materials and dynamically deformable substrates will increase the utilization and importance of these techniques in various fields, including cell research and biosensors.

## 1. Introduction

Substrates play a crucial role in bioengineering by providing a biomimetic environment [1,2,3,4] and by enhancing various biological functions through interactions with biological materials [5,6]. Various polymers and hydrogels are widely used as substrate materials, including polyethylene (PE) [7], polypropylene (PP) [8], polytetrafluoroethylene (PTEF) [9], polymethylmethacrylate (PMMA) [10], polydimethylsiloxane (PDMS) [11,12], collagen [13], fibrin [14], and Matrigel [15]. These materials can be categorized into soft and hard substrates based on their physical and chemical properties and can be mixed to create hybrid substrates for improved functionality. To maximize the performance of these substrates, it is essential to design them while considering their interactions with biological materials. In particular, the surface topography of the substrate, which is in direct contact with biological materials, is a crucial aspect. Precisely controlling the physicochemical properties of the substrate surface can be considered to be a critical challenge in bioengineering. Cell chips (organ chips), a representative example of in vitro biological research, demonstrate that the topography and properties of the substrate surface directly in contact with cells significantly influence cell growth, differentiation, migration, and adhesion [16,17]. For instance, previous studies have shown that cells cultured on substrates with unidirectional topography exhibit a tendency to migrate along the direction of the topography [18,19]. Furthermore, surface modification techniques are also utilized in the field of biosensor applications [20,21]. As the performance of biosensors is largely determined by the interaction between the sensing material and the surface, optimizing the physicochemical properties of the sensor surface is of paramount importance. Consequently, numerous researchers have endeavored to enhance the sensitivity, selectivity, and stability of biosensors by employing nano/microscale surface topography. Notably, the unique topography formed through surface modification techniques is highly advantageous for improving biosensor performance, as it maximizes the contact area with the sensing material and enables precise control of surface properties. Therefore, optimizing the topography in accordance with the research objectives is a prerequisite, and surface deformation techniques are utilized to modify the surface topography for this purpose.

Surface topography can be fabricated in various forms through physical [22] and chemical [23] modifications. This review primarily focuses on surface deformation induced by physical modification. Distinctive topographies such as wrinkles [24], creases [25], and ridges [26] are commonly employed, and these surface deformations are formed by the interaction between the elastic energy and surface energy of the substrate. To induce surface deformation, methods that apply compressive stress to the substrate are mainly used, resulting in diverse patterns depending on the substrate’s material properties, geometric structure, and loading conditions. This review highlights the biological applications that utilize substrates enhanced through the modification and optimization of surface topography. The first part provides a comprehensive overview of the types of surface deformations that cause changes in surface topography and the techniques used to form them. In addition to the techniques, various substrates are categorized into hard, soft, and hybrid materials that integrate both, and their characteristics are summarized. The second part explores a wide range of biological applications utilizing surface deformation, with a primary focus on cell studies, where surface deformation is most actively utilized, along with other examples such as biosensors. Finally, the review discusses the future directions and prospects for research in the field of surface deformation.

## 2. Surface Deformation Techniques with Various Biocompatible Substrates

### 2.1. Biocompatible Substrates

In the realm of biological research, particularly in studies where surface deformation plays a crucial role, the selection of a biocompatible substrate is vital. Ideally, such a substrate should easily undergo surface deformation, meaning its surface topography is amenable to significant alteration by external influences. Beyond the biocompatible substrates previously mentioned, such as polydimethylsiloxane (PDMS) and polymethyl methacrylate (PMMA), other materials like glass and graphene also serve as viable biocompatible substrates in biological research. Typically characterized by their initially flat surfaces, these substrates require the application of various physical or chemical external factors to create microstructures. The specific methods employed for microstructure fabrication vary depending on the substrate, attributable to each material’s unique physical properties. Consequently, substrates can generally be divided into two categories, namely soft and hard substrates, each necessitating different techniques for microstructure development.

#### 2.1.1. Soft Substrates

Soft substrates are the widely used biocompatible substrates and include polymers, foams, gels, colloids, and others [27,28]. In addition, biologically derived materials are almost all soft substrates. The main characteristic of soft substrates is that they change shape easily due to external factors. They are easily compressed, cut, bent, and stretched. In other words, the structure and shape of soft substrates are easily deformed because of the low binding energy between their molecules [29,30,31]. The polymers represented by PDMS and PMMA are widely used as building blocks in the entire field of biological research due to their low cost, quick and easy prototyping, and, above all, their high biocompatibility. Among soft substrates, hydrogels occupy a very special place. Hydrogels are generally hydrophilic polymeric materials with a high-water content, and they are also found in vivo [32]. Hydrogels are a major component of the extracellular matrix (ECM), and the ECM plays a key role in the organization of cells and tissues [33]. Typical naturally occurring hydrogels include collagen, matrigel, gelatin, and fibrin, all of which are major components of the ECM. This suggests that hydrogels are the most suitable materials that can be utilized to create the most in-vivo-like environment in an in vitro system. In addition to these naturally occurring materials, artificially synthesized hydrogels also exist, and, depending on their specific properties, they can be used in a variety of studies. However, these soft substrates have the limitation of relatively low mechanical properties such as electrical and thermal conductivity [34]. This is a major drawback that limits the utility of in vitro systems based on soft substrates, making it difficult to utilize soft substrates for applications such as electronic sensors, devices, and energy harvesting.

#### 2.1.2. Hard Substrates

Hard substrates are materials that have the opposite properties of soft substrates, with high binding energies between molecules that typically break the original structure when deformed [35]. Examples include ceramic, metal, graphene, nanoclay, carbon nanotube, metal chalcogenide, and glass. They are characterized by being relatively difficult to deform, but very stable in shape. These hard substrates have relatively limited applications in biological research because their inherent physical properties are significantly different from those of the human body. However, they have high electrical and thermal conductivity, so they can be utilized in biosensors and antimicrobial applications where these two properties are required [36,37]. One example is graphene, which has gained prominence in recent years in biological and biomedical applications due to its unique properties: thin, flexible, and yet harder than a diamond. Recent studies have demonstrated that graphene can be used to guide cell behavior, including cell proliferation, viability, osteogenesis, and adhesion [38,39]. However, outside of these special cases, hard materials have relatively limited applications in tissue engineering or as cell substrates due to their difficult surface modification, low solubility, and physical properties that differ significantly from in vivo systems.

#### 2.1.3. Hybrid Substrates

Recently, studies have been published that utilize substrates that are a blend of these two materials, and these blends of soft and hard substrates are referred to as hybrid substrates [40,41]. They are usually utilized in the form of a double layer of soft substrate on top of a metal film, which is a type of hard substrate. Hybrid substrates have recently gained attention in the field of biological research because they utilize the advantages of both soft and hard substrates (complementing each other’s limitations). As the activity and proliferation of cells have been verified in studies using composite materials with high electrical and thermal conductivity and high biocompatibility through the combination of suitable materials, they have begun to be widely used in various studies.

Depending on the properties of these materials, they can be categorized into soft, hard, and hybrid substrates, and their advantages and limitations are summarized in Table 1.

### 2.2. Surface Deformation Examples

**Figure 1:**
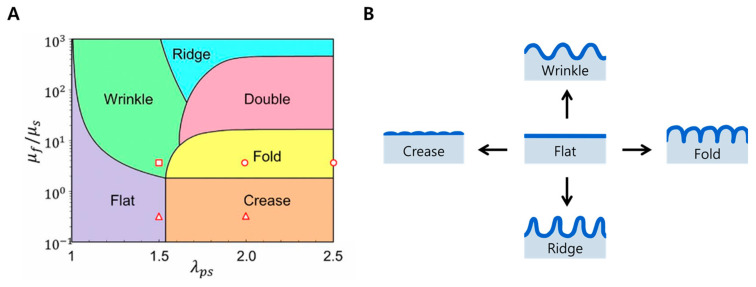
Types of various surface deformations. (**A**) A phase diagram for predicting the formation of various deformation patterns. The circular markers indicate fold condition, the square marker indicates wrinkle condition, and the triangular markers indicate crease condition. Reprinted from [113] under the Creative Commons CC BY-NC-ND license, (**B**) examples of the different surface deformations discussed in this review. Since double (or period double) is rarely used in the biological applications covered in this review, it is considered a type of fold and is not described separately.

In general, surface deformation refers to a change in the shape of a surface due to external factors which can be caused by physical and chemical factors. These deformations are different from programmed techniques (e.g., soft lithography) that transfer a conventionally designed pattern onto a material surface, as they are spontaneous deformations that occur due to changes in internal stress distribution caused by external factors rather than an intended shape (Table 2). Typical external factors include physical factors such as tension, compression, bending, and twisting. Surface morphology changes are based on the formation of surface microstructure. The resulting surface deformation, which causes surface instability, can be categorized into wrinkle, crease, fold, and ridge depending on its morphological characteristics (Figure 1).

#### 2.2.1. Wrinkling

**Figure 2:**
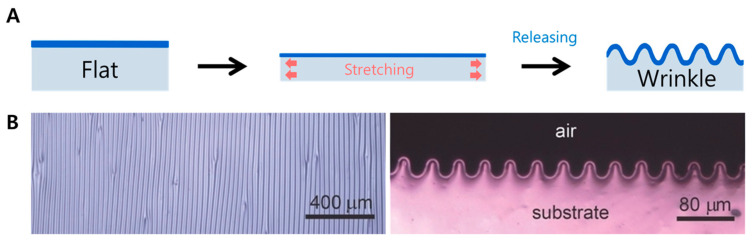
Wrinkling formation by stretching and releasing. (**A**) Process, (**B**) real wrinkling surface. Reprinted with permission from [118].

Wrinkling is the most general form of surface deformation (Figure 2). The specific characteristics of the wrinkles are the infinitesimal amplitude of the surface deformation and broad surface coverage [114]. The external, unidirectional stretching is required to form a wrinkled surface. After the releasing, then a regular wavy structure is formed. Commonly, when the base material is clearly divided into two or more layers, deformation occurs via this external stretching. Wrinkling can also be fabricated by other techniques rather than stretching, such as compression [115] and solvent evaporation [116]. Also, the temperature-triggered shape recovery forms the wrinkled surface [117]. When the base material is a homogeneous polymer, such as a hydrogel, volume change due to swelling also causes a wrinkling structure. The strain location of the gel is also key. Polymerization of PDMS on thin PS film causes the thermally induced volumetric shrinkage to form a natural wrinkled surface. For gradient wrinkle, a prism mask causes a shaded plasma oxidation on elongated soft substrate. After the release, increasing wrinkles are revealed. In most cases, wrinkling can be considered a soft substrate, meaning that it does not use separate patterning or techniques such as lithography or etching to induce deformation by causing surface deformation.

#### 2.2.2. Creasing

**Figure 3:**
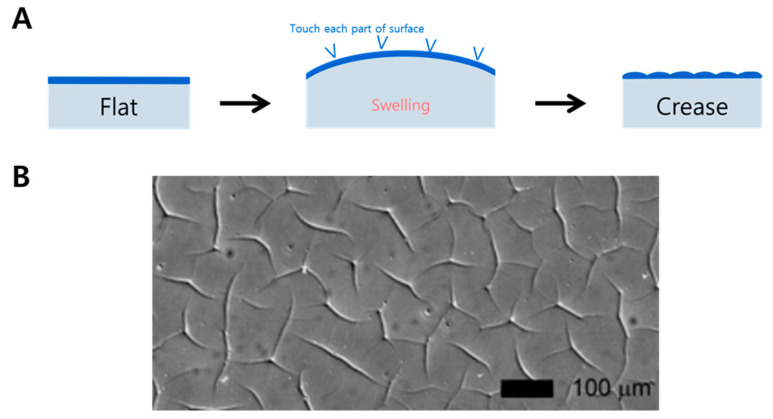
Creasing formation by swelling. (**A**) Process, (**B**) real creasing surface. Reprinted with permission from [122].

The creasing structure has been observed on elastic materials compressed by mechanical force [119], constrained swelling [120], and temperature change [121]. Thus, similarly to wrinkling, the creation of creasing also primarily utilizes soft substrates. The main characteristic of a creasing structure is the presence of sharp indentations on the surface (Figure 3). A representative example in nature is the cerebral cortex. In general, surface creasing is the result of solvent absorption. The key is a large swelling, then, the parts of the surface begin to touch each other, forming localized sharp folds, a pattern called creasing. Also, sometimes the furrows of the substrate are pulled to form a creasing surface. Different pressures form the different reproducible creased surfaces. The soft materials show reversible contraction and expansion following stimulation, such as temperature or a specific wavelength of light.

#### 2.2.3. Folding and Ridge

**Figure 4:**
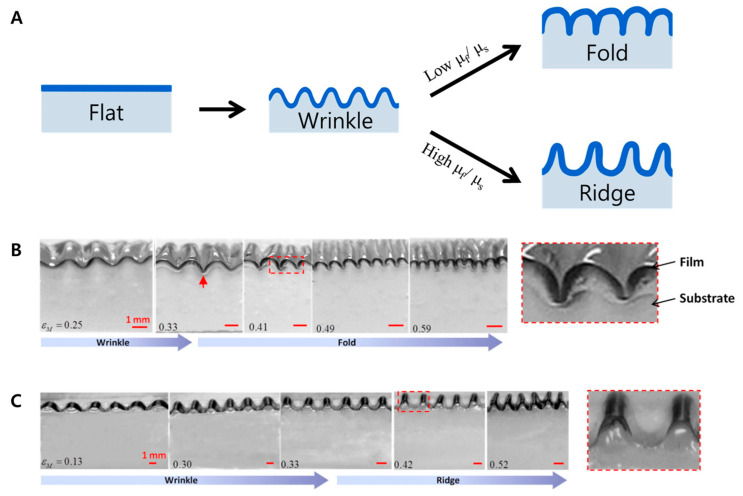
Folding and ridge formation by compression based on the modulus ratio (μ_f_/μ_s_). (**A**) Process, (**B**) real fold surface (The red arrow indicates the valley, a characteristic point that marks the bifurcation of the wrinkle and fold), (**C**) real ridge surface. Reprinted from [22] under the Creative Commons CC-BY license.

Surface folding and ridge formation can be considered to be secondary surface deformations (Figure 4). In general, surface folding and ridge formation have been observed in hybrid materials with polymer films and substrates [22]. When the mismatch strain is applied to the hybrid materials, as a first step, the wrinkling structure is formed. As the strain further increases, the structures of the materials bifurcate into more complex patterns. The folding and ridges form more complex patterns; therefore, we can consider those as secondary surface deformations. The standard of the bifurcation is the modulus ratio (μf/μs) between the polymer film (μf) and the substrates (μs). When the modulus ratio (μf/μs) is low (relatively compliant film), the folding is formed. When the materials are compressed at the onset of wrinkling, the wrinkling loses the initial sinusoidal, harmonic pattern. As a result, localized folding is formed, while the relatively high μf/μs engenders ridge formation. The key characteristics of a ridge include a larger amplitude and a smaller wavelength, forming a high-aspect-ratio pattern. Put simply, when the wrinkling structure no longer exhibits a sinusoidal wave pattern due to the application of additional force, it becomes a ridge. The various standard organs that require a large surface area like the stomach and intestine are commonly composed of folded mucosa. To mimic this in vitro, the stretchable, tough hydrogel-like polyacrylamide and alginate have been pre-stretched by bonding them to a PDMS holder [113]. The rigid surface formation is simply the result of a pre-stretch of the substrate followed by its release. Computational simulations have been conducted to find the proper wavelength before the experimental formation. Whether a thick, soft substrate is stretched uniaxially [123] or in two orthogonal directions [124] depends on the purpose. A stiff film layer composed of gold [124], stiff PDMS (5:1 mixing ratio) [123], and GelMA (gelatin methacrylate) film [113] is often attached to the surface to decrease the strain. An extremely curvy shape mold is fabricated by photolithography to replicate rigid surfaces.

## 3. Biological Applications

### 3.1. Surface Deformation for Cell Studies

Surface wrinkling topographies are mostly used as surface patterns for biological studies, mainly due to their ease of fabrication and tunability. Wrinkled topographies are not only used for promoting cell adhesion and differentiation, but also for migration and guided contact studies for various cell lines including endothelial and stem cells [125,126,127,128], fibroblasts [129,130,131,132], and neurons and myocytes [133,134,135,136,137].

#### 3.1.1. Cell Differentiations

**Figure 5:**
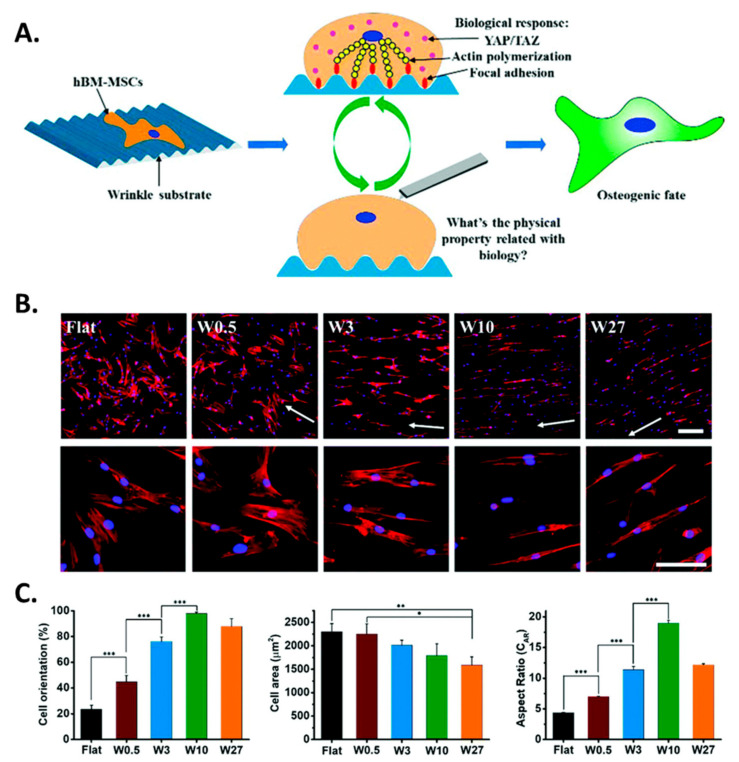
Image (**A**) shows that the hBM-MSCs cultured on the winkled surface could be stimulated into osteogenic differentiation. Image (**B**) shows the analysis of different wrinkle sizes on the morphology of the hBM-MSCs. The microscopic images illustrate the cells cultured on the different surfaces with different surface topography. Cell cytoskeleton and nucleus were stained using TRITC-labeled phalloidin (red) and DAPI (blue). The white arrow shows the direction of the wrinkles (Scale bar for all images is 100 μm). Image (**C**) shows the analysis of cell orientation, cell area, and cell aspect ratio (CAR) for different surface topographies (Data are shown as mean ± standard deviation (SD), and * *p* < 0.05, ** *p* < 0.01, *** *p* < 0.001). Reprinted from [141] under the Creative Commons CC-BY license.

For example, studies have revealed that topography has a significant role in the cell stiffness of human bone-marrow-derived mesenchymal stem cells (hBM-MSCs), causing osteogenic differentiation [138]. Wagner et al. compared the cell interaction on the PDMS wrinkled surface to a flat surface and showed higher osteogenic differentiation [139]. Similarly, Mrksich et al. showed that cells cultured with an increasing aspect ratio of the surface pattern show an increasing osteogenic differentiation [140]. Recently, Yang et al. showed that cell elongation is associated with physical properties such as cell stiffness along with other biological properties. A PDMS-based wrinkled surface fabricated through a single-cell mechanical alteration has been analyzed using atomic force microscopy (AFM) and it has been found that cell stiffness is another major factor when it comes to the osteogenic differentiation of stems cells [141], as shown in the Figure 5.

**Figure 6:**
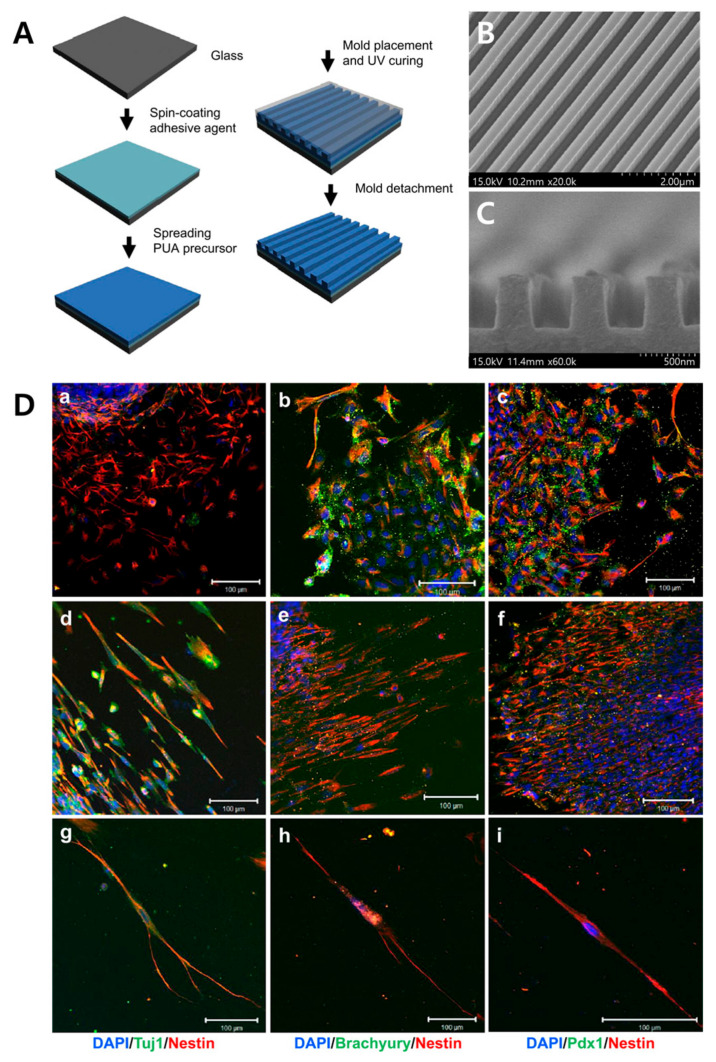
Image (**A**) shows the fabrication of a nanoscale ridge pattern array using UV-assisted capillary force lithography. Image (**B**) shows the SEM image of the PUA ridge-patterned surface. Image (**C**) shows a cross-sectional SEM image of the substrate. Image (**D**) shows the neural differentiation of hESCs cultured on the flat surface and on the ridge surface. The cells were immunostained with DAPI, tuj1, brachyury, PDX1, and nestin. Images (**a**–**c**) show the cell differentiation on flat surface for a five-day culture, images (**d**–**f**) show the cell culture on the ridge surface over 5 days, and images (**g**–**i**) show the culture over 10 days on the ridge-patterned surface (Scale bar for all images is 100 μm). Reprinted with permission from [142].

Human embryonic stem cells (hESCs) are pluripotent cells with potential applications in biomedical and tissue engineering. hESCs neuronal differentiation has been studied using different biochemical and biological agents, but these agents are associated with a high cost and require complex handling to obtain the optimal concentration for proper differentiation without side effects. Therefore, Lee et al. studied the hESCs differentiation using the nanoscale ridge/groove surface pattern arrays without using any differentiation-inducing agents [142]. Briefly, the 350 nm controlled nanoscale ridge/groove pattern was fabricated using UV-assisted capillary force lithography. The hESC cells were seeded on both flat and 350 nm ridge/groove patterned surface and cultured for five days. The cells were stained with tuj1 (immature neuronal marker), brachyury (mesoderm marker), PDx1 (endoderm marker), and nestin. Flat surface cells were found to be randomly spread, whereas the cells on cultured on the 350 nm ridge/groove patterned surface were found to be aligned in the direction of the pattern. Moreover, the ten-day culture of cells on the 350 nm ridge/groove patterned surface revealed a well-structured neurite extension along the direction of the ridge/groove pattern, as shown in the Figure 6.

#### 3.1.2. Wound Healing

**Figure 7:**
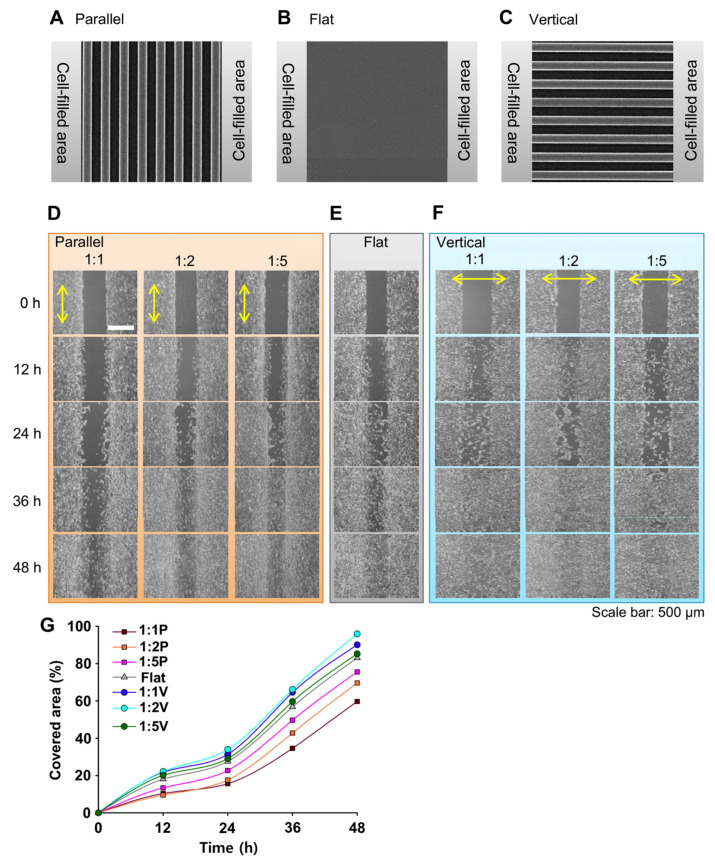
Images (**A**–**C**) show representative parallel and vertical topographies together with the flat control (no pattern on the PUA surface). Images (**D**–**F**) show time-lapse microscopic images of in vitro wound healing with respect to the orientation and densities of ridge/groove topography. The yellow double arrow indicate orientation of the patterns. Image (**G**) shows fibroblast coverage, which was quantified for each topographical orientation and density. The results indicated that the perpendicular topography exhibited significantly higher coverage rates in comparison to the smooth control surface and the topography with parallel alignment. Reprinted with permission from [18].

Another application of ridge/groove patterned structures for cell study revolves around the dermal wound healing process. Kim et al. fabricated the synthetic extracellular matrix scaffold which had uniformly spaced nanogrooved surfaces [18]. The nanopatterned surfaces were fabricated using the UV-assisted capillary molding technique. The surface topography showed vertical or parallel orientation using nanogrooves with three different spacing ratios of 1:1, 1:2, and 1:5. The cultured NIH-3T3 cells showed clear differences with patterns, and the patterns guided the cell migration orientation in the wound healing process. These patterns mimic the well-aligned structure exhibited by collagen fibers in in vivo dermis, and this study effectively validated the effect of the orientation and density of these patterns on cell migration (Figure 7).

#### 3.1.3. Cell Alignment

**Figure 8:**
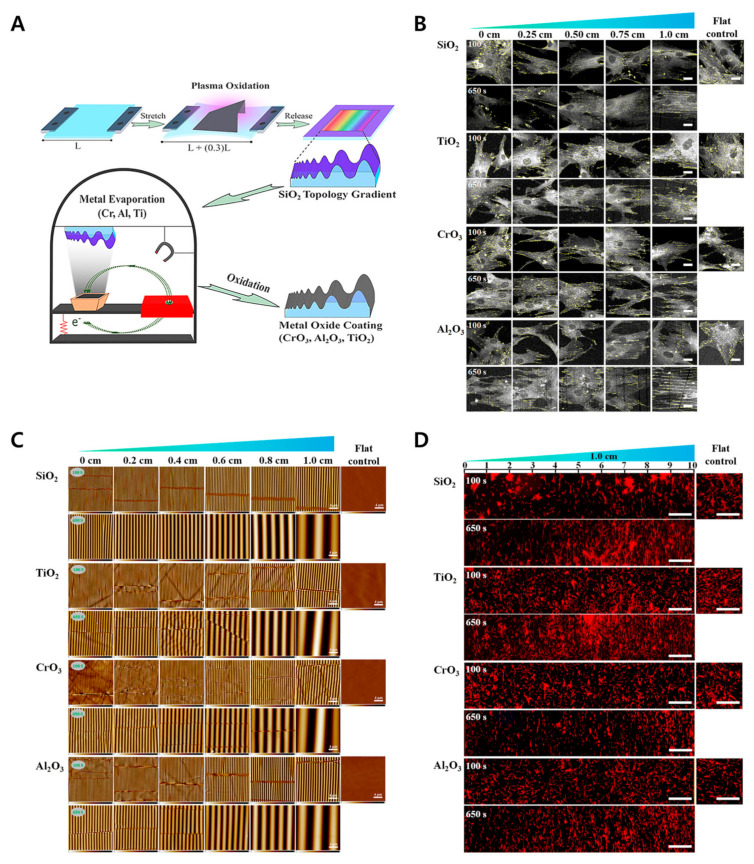
Image (**A**) shows the schematic process of fabricating the winkled gradient device using the plasma oxidation process and then air exposure under ambient conditions. (**B**) Focal length images of the cells after two days of culture (Scale bars = 22 μm). (**C**) AFM images of surface topography with different metal oxides (Scale bars = 4 μm). (**D**) Fluorescence images of the cells along the full length of the device (Scale bars = 1 mm). Reprinted from [147] under the Creative Commons CC-BY-NC-ND license.

**Figure 9:**
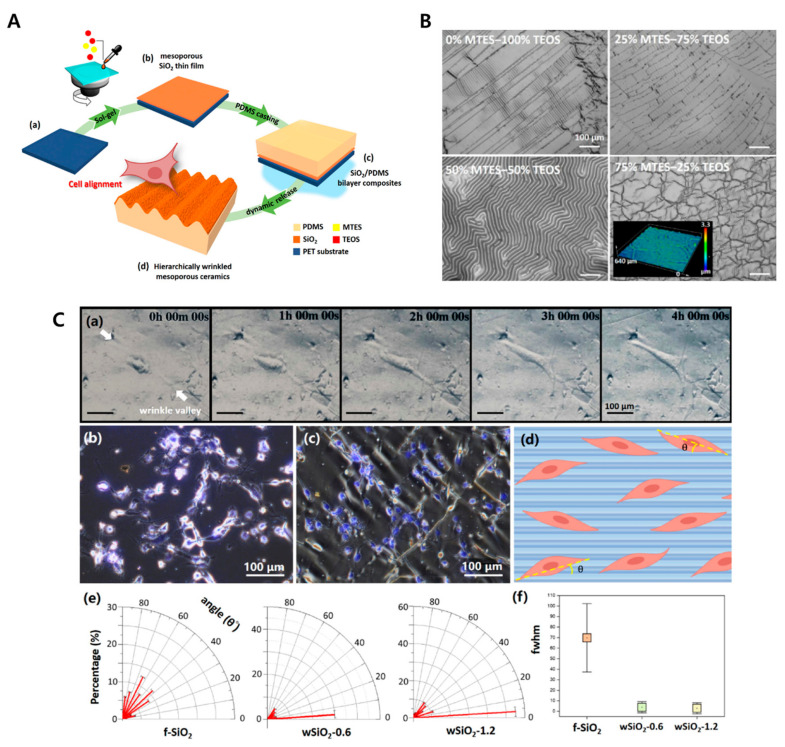
Image (**A**) shows the schematic illustration of hierarchically wrinkled mesoporous ceramics platform fabrication using sol-gel reaction for cell alignment. The sol-gel precursor is coated on the cleaned PET substrate and, subsequently, PDMS casting is performed; finally, the dynamic interference release and flipping of PDMS/SiO_2_ generates the hierarchically wrinkled surface. Image (**B**) shows the optical images of the surface synthesized using sol-gel precursor mixtures with different weights of MTES:TEOS. Image (**C**) shows cell images and analysis results. (**a**) Optical images of blood vessel cells on the wrinkled surface. The white arrows indicate wrinkle valley. (**b**,**c**) The fluorescence images show the comparison of cell alignment on the flat surface and the wrinkled surface. (**d**) Illustration of cell growth angles (θ°) and wrinkle orientation. (**e**) The angular histogram of cells on the surface was measured. (**f**) The FWHM of cell alignment distribution. Reprinted with permission from [148].

In biological environments, cellular and acellular components are anisotropically organized and aligned in specific directions, providing the structural and mechanical properties for actuating biological functions. Many studies have developed a platform for anisotropic or isotropic patterns to analyze the organization, alignment, and development of various cell types [143,144,145,146]. However, these polymeric substrates are limited in their use in biological applications such as dental and orthopedic studies, which mostly requires inorganic materials. Studies have been able to fabricate the inorganic biological materials for cell contact and guidance. For example, Zhou et al. reported a multiscale topographical gradient approach with different inorganic materials with PDMS wrinkled surface [147]. Briefly, the platform was prepared by plasma oxidation, and the human bone-marrow-derived mesenchymal stem cells (hBM-MSCs) were seeded, inducing the cell alignment along the wrinkle direction as shown in Figure 8. Recently, in another study, Xie et al. utilized sol-gel reaction and surface wrinkling from elastomer crosslinking contraction to fabricate the combined ceramic platform [148], as explained in the Figure 9. This method provides controllable geometric dimensions and allows for the culture of human blood vessel cells.

#### 3.1.4. Dynamic Stimulus Platform

**Figure 10:**
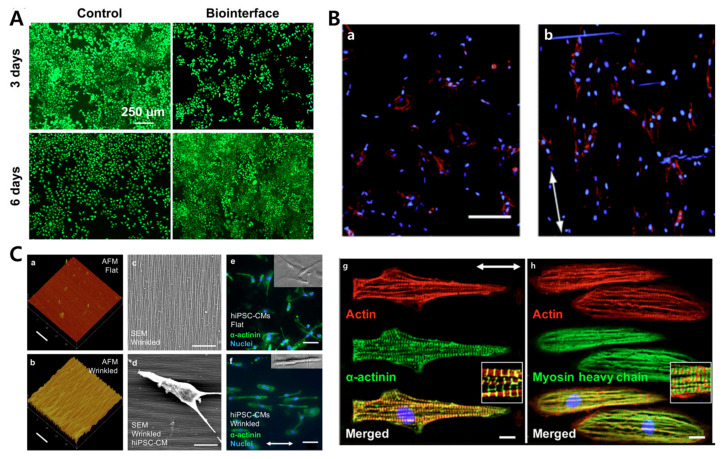
(**A**) WST-1 assay analysis showing the cell metabolic activity on the biointerfaces compared to the control surface; after proliferation over 6 days, it increased by 25%. Reprinted with permission from [150]. (**B**) Effect of the wrinkled surface on cell alignment. The cells were analyzed with phalloidin (cytoskeleton) and DAPI (nuclear) assays, with red showing the cytoskeleton and blue showing the nuclear material. The figures show that cells on the wrinkled surfaces (**b**) exhibited alignment compared to the cells on the flat surface (**a**). The white arrow indicates wrinkle orientation (Scale bar = 200 μm). Reprinted with permission from [151]. (**C**) Alignment of hiPSC-CM cells on the static SMP-PEM substrate. (**a**,**b**) The figures show the AFM image of flat and wrinkled surface(Scale bars = 5 μm). (**c**,**d**) The figures show SEM images of a wrinkled surface and cell (Scale bars = 10 μm). (**e**,**f**) The fluorescent images show randomly orientated cells and highly aligned cell on a flat surface and wrinkled surface, respectively (Scale bars = 50 μm). The white arrow indicates the wrinkle direction. (**g**,**h**) Further, the images also show the well-aligned myofibril structure on the wrinkled surface. The white arrow indicates wrinkle direction (Scale bars = 10 μm). Reprinted with permission from [152].

Dynamic stimulus from external microenvironment or extracellular matrix regulates the cell differentiation, morphology, and functions. However, most of the platforms used for cell studies are static platform and do not provide dynamic stimuli over the experimental time. Shape memory polymers (SMPs)-based cell culture platforms can provide dynamic changes in surface morphology to mimic the cellular environment. Davis et al. developed a temperature responsive topography platform using SMPs, the platform changed surface topography under cell culture temperature conditions, and, hence, induced the morphological changes in the embryonic fibroblast cells [149]. Further, Bernardeschi et al. utilized the SMP-based dynamic platform for mechanobiological cell culture studies. Briefly, wrinkles were formed on a 90 nm thick conductive layer over a PDMS flat surface, and the cell adhesion and proliferation of human neuroblastoma was analyzed [150]. Similarly, Yang et al. fabricated a flat-to-textured surface transition platform by coating SMP on a gold film. Under compressive bulking of the thin gold film, the dynamic nanoscale wrinkles affected the cell nuclei orientation and cell motility [151]. More recently, Sun et al. studied the cellular process involved in cardiomyocyte (CM) response to dynamic environmental changes. An SMP-based flat-to-wrinkled transition surface under a temperature change was fabricated and cell morphological and intracellular myofibril reorganization under the dynamic topography changes were analyzed [152]. Figure 10 shows images from these studies.

### 3.2. Molecular Study

Surface deformation is not only used for cell study, but also for various molecular studies depending on the type of material and deformation. Sensors, able to render the surface superhydrophobic, and the nanoparticle array are the typical examples of surface deformation applied to biological studies.

#### 3.2.1. Biosensors

**Figure 11:**
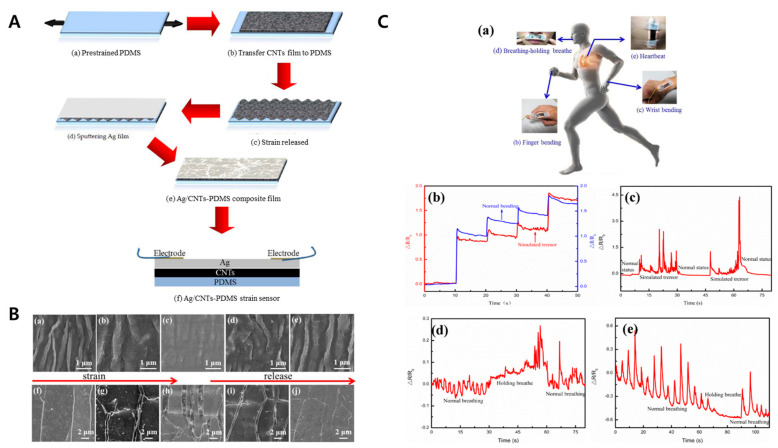
(**A**) Schematic figures for the fabrication of Ag/CNTs-PDMS film sensor. A thin CNT film is transferred to PDMS, and the pre-strain and release strain generate the wrinkle structure. Subsequently, the Ag film sputters and the electrodes are assembled for flexible and wearable sensor. (**B**) SEM image showing the morphology of a wrinkled structure according to the strain strength. (**a**–**e**) Original CNTs, (**f**–**j**) Ag/CNTs-PDMS composite films at tensile strain 0%, 5%, 10%, 5%, and 0%, respectively. (**C**) Film sensor monitoring of weak human body signals. (**a**) Sensor’s location overview. Signals from (**b**) finger bending, (**c**) the wrist, (**d**) the upper lip, and (**e**) the chest. Reprinted from [153] under the Creative Commons CC-BY license.

High-performance biosensors can be fabricated by applying the surface deformation technique to the metal–soft-material bilayer. Yang et al. developed a sensor using wrinkled Ag/CNTs-PDMS film, which can measure weak signals such as tremors, breath depth, and the corresponding heartbeat response with the single monitoring of the human body. This sensor, based on hybrid materials, not only enables the diagnosis of Parkinson’s disease, but also predicts various other syndromes [153]. Figure 11 shows images from this study. Oyunbaatar et al. demonstrated that a PDMS-based cantilever sensor coated with a Ti/Au thin film can effectively monitor the cardiac cell behaviors by measuring the displacement of the cantilever. Compared with the bare cantilever, the wrinkled cantilever showed a much higher displacement and a longer culture period. This cantilever system could be the excellent route for analysis of the cell responses to external stimuli [154]. Tang et al. investigated the highly stretchable sensor. Silver, a widely used electrode material, was used and good electrical conductivity was maintained even in a high-strain environment. Furthermore, this electrode was applied to wearable wireless sensors which can sense the strain from the bending of a human finger [155].

**Figure 12:**
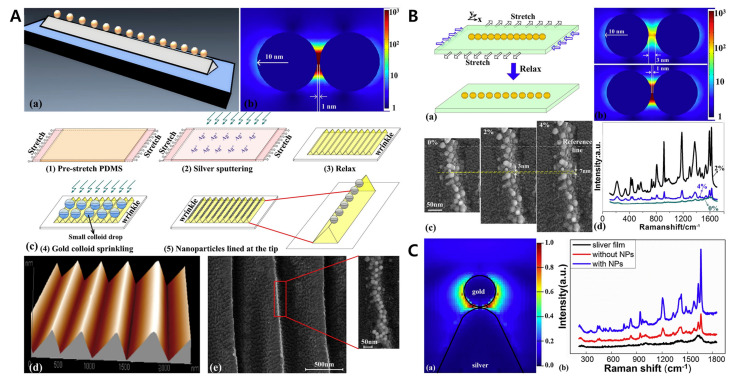
(**A**) Schematics for the fabrication of a nanoparticle array at the top of the ridged crack. Image (**a**) shows ordered nanoparticles at the tip of wrinkled structure. Image (**b**) shows electromagnetic field simulation of two nanoparticles. Image (**c**) shows manufacturing process. A silver nanofilm is sputtered on the pre-stretched PDMS. By releasing the films, triangle cross-section wrinkles are formed. Colloid drops are located on the substrate and only gold nanoparticles remain after evaporation. (**d**) AFM and (**e**) SEM images show the aligned nanoparticles at the top of the wrinkled structure. (**B**) The gap between two nanoparticles can be tuned by (**a**) mechanical stretch and release force. (**b**) XFDTD simulations show that the electromagnetic effect is controlled by the nanogap. Image (**c**) shows SEM image of a nano-gap under different stretching forces. (**d**) The nanogap is determined using applied stress and SERS intensity is maximized at 2% stretching condition. (**C**) Electromagnetic field is maximized (**a**) between gold nanoparticle and silver substrate. (**b**) The SERS effect enhanced the wrinkled structure under the nanoparticle condition. Reprinted with permission from [156].

Nanoparticles have a large surface-to-volume ratio, which increases the effect of the material surface compared to macrosized materials. Thus, nanoparticles can change the physical and chemical properties of the materials. Therefore, various research such as the development of measurement devices, DNA detection, protein detection, and drug development has been conducted on nanoparticle array technology. Surface wrinkling technology enables the formation of relatively simple nanoparticle arrays. Tang et al. succeeded in forming an array of single nanoparticles using wrinkled PDMS film. Silver was sputtered on the PDMS surface, forming the triangle cross-section of the surface ridge. The nanoparticles were lined at the ridge tip and the SERS effect was enhanced by using hybrid materials. Single nanoparticles can be detected using this wrinkle PDMS platform even with a significantly low limit of detection level (~10–20 M) [156,157] (Figure 12). Park et al. developed a PDMS/gold nanoparticle (AuNPs) substrate to show the process of AuNPs synthesis on the ridged crack. After fabricating the wrinkle through a stretch–release process, the silica layer cracking part was formed though oxygen plasma treatment, with SEM images showing the AuNPs specifically arranged at the top of the cracked region. This substrate can be applied to biosensors or optical devices [158]. Recently, Gole et al. showed AuNPs self-assemble on the wrinkled PDMS–graphene substrate. The distance between nanoparticles was successfully controlled by regulating the molecular weight of the polymer brush. Moreover, a polarized absorption spectra analysis showed the applicability to electrons and sensors [159].

#### 3.2.2. Surface Wettability Control

**Figure 13:**
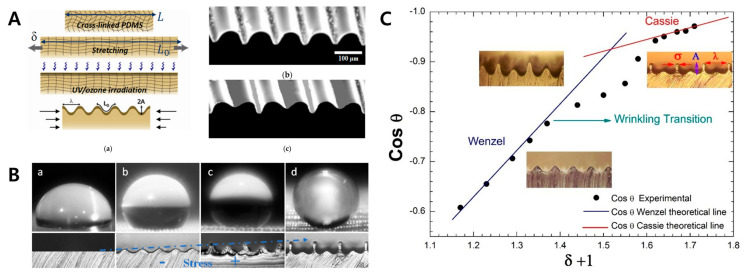
(**A**) Schematics for the fabrication of a micro pattern on the PDMS substrate. (**a**) Stretching, UV/ozone treatment, and the releasing process can generate surface deformation patterns. (**b**,**c**) The SEM image shows a constant wrinkle pattern. (**B**) Water contact angle measured under various surface condition. (**a**) On the flat surface, the contact angle is 110° and, (**b**,**c**) as the stress increases, the contact angle also increases. (**d**) Finally, in the cassie state, the contact angle reaches close to 180°. The blue line indicates increased height of wrinkled structure. (**C**) Black points show the experimental results of surface deformation corresponding to the theoretical graphs. Reprinted from [160] under the Creative Commons CC-BY license.

Surface deformation can be used to control the surface wettability and create super-hydrophobic surfaces. Sabbah et al. realized a superhydrophobic surface with a contact angle of 150 degrees using a simple surface deformation technique. Wrinkled and ridged surfaces were created through compression stress control. As the strain and the surface deformation increased, the contact angle also increased, resulting in a superhydrophobic surface with a contact angle close to 180° [160] (Figure 13). Jun et al. reported a high-performance wrinkled actuator, namely wrinkled dielectric elastomer actuators (WDEAs). Surface wettability of actuators can be controlled by applying electrical stimuli to the carbon black electrode. In addition, a change in the direction of wettability is possible, so it can be applicable to various flexible actuator fields [161]. Lu et al. designed the ridged surface with tunable wettability. Cracked Zn/PDMS surface enables reversible wettability control over a wide range of contact angles, i.e., 115°–143° [162].

#### 3.2.3. Antibiofouling Film

**Figure 14:**
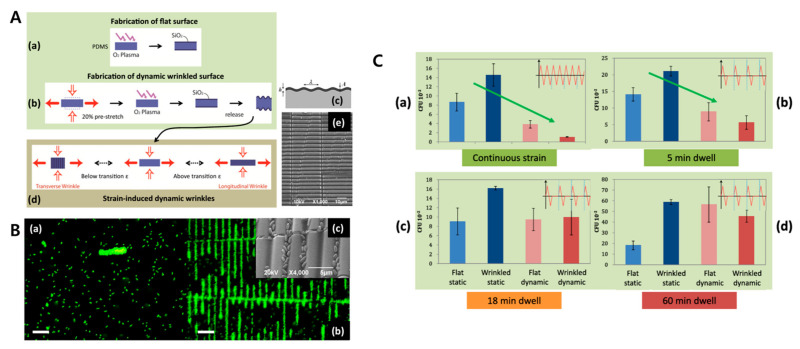
(**A**) Schematics for the fabrication of wrinkled PDMS. Image (**a**) shows plasma treatment without applied strain. In contrast, to form a uniform wrinkle structure, (**b**) a20% pre-stretch, oxygen plasma, and releasing sequence were applied. (**c**) Schematic image of highly controlled and regular buckling of the surface. Image (**d**) shows strain-induced dynamic wrinkles. (**e**) The SEM image shows that the wrinkle patterns are well formed on the PDMS surface. (**B**) Fluorescence microscopy image showing (**a**) the attached *P. aeruginosa* PA-14 bacteria forming a pattern at the valley of the wrinkle, (**b**) while bacteria appear to be randomly distributed on the flat PDMS. (**c**) SEM image of bacteria on the surface features. (**C**) Compared to a static culture, a dynamic vibration condition drastically inhibits bacterial attachment. However, introduction of dwell time weakens the effect of the vibration. (**a**) Continuous strain condition, with no dwell time. (**b**–**d**) Introduction of dwell time of 5, 18, 60 min between the strain cycles. Reprinted from [163] under the Creative Commons CC-BY license.

In addition to what has been described above, surface deformation has been widely applied in various biological fields. Zhang et al. created a marine antibacterial surface by adding polymer brush to a wrinkled PDMS surface. The adhesion of marine organisms is greatly influenced by the topography of the surface and the antibiofouling effect of a wrinkled surface has been demonstrated. In particular, the POEGMA (Poly(ethylene glycol)methacrylate) brush and PSPMA (Poly(3-sulfopropyl methacrylate potassium salt)) brush showed the least contamination [64]. Epstein et al. reported on a wrinkled biofilm to prevent or promote bacterial contamination without any chemical or drug treatment inspired by some marine organisms. In a static culture, the bacterial attachment level was improved on the wrinkled surface. However, when vibration was applied, the bacterial attachment was significantly reduced, and the maximum effect attained was under the 20% dynamic amplitude condition [163]. Figure 14 shows images from this study. Gilmore et al. observed the lipid bilayers mechanochemical coupling reaction changing according to the real-time wrinkling. As the surface deformation progressed, the lipid bilayer pole localization was observed. This result corresponded to the membrane protein response in vivo [164].

In addition to these representative examples of molecular studies, a more comprehensive set of previous studies can be found in Table 3.

## 4. Conclusions and Outlook

This review covered a wide range of substrates, categorized as soft, hard, and hybrid, examined the surface deformation techniques that can be utilized in relation to the three types of substrates, and, finally, covered a wide range of biological applications in an integrated manner. Thus, the novelty and importance of this paper is that it provides integrated insights.

In cell-based research, surface deformation of soft substrates, especially wrinkle structures, is widely utilized in various studies of cell behaviors, such as cell adhesion, differentiation, and migration. Notably, it has been shown that the physical characteristics of wrinkle structures play a crucial role in osteogenic differentiation of stem cells and neuronal differentiation. Furthermore, it has been verified that nanosized ridge topography can guide the direction of cell migration, a phenomenon which can be used as part of the wound healing process.

On the other hand, in the fields of biosensors and biofilms, surface deformation techniques have been mainly utilized based on hard or hybrid substrates. Surface deformation techniques play a key role in various biological studies, such as high-performance biosensors using metal–soft-substrate bilayers, self-assembly of nanoparticles using wrinkled cracks, control of wettability through wrinkle structures, and prevention of marine biofouling.

In the future, it is expected that the commercialization of hybrid materials, which are cost-effective and highly versatile, will be actively pursued over the usage of soft or hard substrates alone. Indeed, commercialization is already underway in cell/organ chips field, and it is anticipated that commercialization in various other biological fields will be realized soon. Furthermore, recent research results have shown reversible deformation [165] or transformations into various structures with controllable shapes [166], not limited to the existing fixed-structure substrates. To discuss dynamically deformable substrates in more detail, existing technologies have been used, mainly, to produce irreversible surface deformation and conduct experiments on fixed surface topography. In order to study a more diverse range of surface deformations, different condition values have been applied to cause different surface deformations through multiple experiments, an approach which has proved to be cumbersome. However, new materials and technologies that can produce different topographies on the same surface can pave the way for breakthroughs in this field by dramatically reducing the time and cost associated with existing research. Furthermore, we can also discuss extending the research to smart materials that selectively respond to various external stimuli such as temperature, pH, light, and electric and magnetic fields to change their surface topography and function. This could be specifically used for drug delivery, biosensors, actuators, etc., and will enable the construction of more sophisticated and active surface modification systems. Finally, we can also look forward to the convergence with artificial intelligence, which has recently been applied in various fields. AI technologies such as machine learning and deep learning can be used to optimize surface topography and predict properties in advance, enabling a more efficient surface modification. This can significantly improve the efficiency and accuracy of the material development process. If research on the analysis of real-time interactions between biological materials and kinetically deformable substrates based on these new technologies is actively conducted, and if the integration with artificial intelligence technology is further advanced, the applicability and importance of surface deformation techniques are expected to increase further in various fields mentioned in this paper, such as cell-based research or biosensors.

## Figures and Tables

**Table 1:** Summary of representative biological materials in terms of key features and applications.

Type	Benefits	Limitations	Materials	Applications
Soft	Low cost, rapid prototyping, high biocompatibility, chemical stability, good mechanical properties, excellent optical transparency, and simple fabrication	Low electrical conductivity and thermal conductivity	PMMA	Cellular assay [42], organ on a chip [43,44], lab on a chip [45], antimicrobial surface [46]
			PDMS	Cellular assay [47,48,49,50,51,52,53], organ on a chip [54,55,56], lab on a chip [57,58,59,60], biomolecular assay, organoid [55]
			Polymers	Cellular assay [61,62], antimicrobial surface [63,64], biosensor [65]
			Hydrogels	Cellular assay [66,67,68,69,70,71,72,73,74,75], organ on a chip [76,77,78,79,80], wound healing [81,82], drug delivery [83,84], organoid [78,82]
Hard	High electrical conductivity and thermal conductivity	Difficult to control the shape of the surface, poor solubility, difference in physical properties from in vivo systems, and opaque surface	Graphene-based materials	Cellular assay [85,86,87,88,89,90,91]
			Metal	Cellular assay [92,93], antimicrobial surface [94], biosensor [95,96,97]
Hybrid	Advantage of both soft and hard materials at the same time and a variety of applications	Difficult to control the shape of the surface, complex fabrication process, and expensive unit price	Graphene–polymers	Cellular assay [40,41,98,99]
			Graphene–glass	Cellular assay [41,100]
			Graphene–hydrogels	Cellular assay [101,102]
			Metal–graphene	Wound healing [103]
			Metal–polymer	Bioactuator [104], lab on a chip [105,106,107]
			Metal–hydrogel	Cellular assay [108]
			Ceramic–hydrogel	Organ on a chip [109]
			Hydroxyapatite–PDMS	Organ on a chip [110]
			Nanoclay–hydrogel	Organ on a chip [111]
			Carbon-nanotubes–hydrogel	Biological transducer [112]

**Table 2:** Comparison of surface deformation and programmed techniques.

Techniques	Methods to Form the Topography	Mechanism
Surface deformation technique	- Physical factors such as tension, compression, bending, twisting, and swelling	- Spontaneous generation due to internal stress distribution changes, mechanical instability - Surface deformation induced by external factors
Programmed technique (e.g., surface lithography)	- Patterning by designed stucture	- Transferring intentionally designed microscopic patterns onto material surfaces

**Table 3:** Summary of molecular studies with surface deformation of substrate.

Application	Surface Deformation	Materials	Reference
Biosensor	Ridge–crack	Metal–PDMS	[153]
	Wrinkle	Metal–PDMS	[154,155]
Nanoparticle array	Ridge	Silver–PDMS	[156,157]
		PDMS	[158]
	Wrinkle	Graphene–PDMS	[159]
Wettability control	Wrinkle, Ridge	PDMS	[160]
	Ridge	Zinc–PDMS	[162]
		VHB-4905–carbon-black	[161]
Biofilm	Wrinkle	PDMS	[64,163]
Lipid bilayer	Ridge	PDMS	[164]

## Data Availability

Not applicable.
